# Elucidating the mechanism of triphenyl phosphate interference in bone metabolism via network toxicology and molecular docking methodologies

**DOI:** 10.3389/fendo.2025.1606877

**Published:** 2025-07-07

**Authors:** Min Xu, Yinxiang Wu, Jiaqi Meng, Mengchen Chen, Chen Ding

**Affiliations:** ^1^ Department of Trauma Orthopedic, First Affiliated Hospital of Naval Medical University, Shanghai, China; ^2^ Department of Pulmonary and Critical Care Medicine, Third Affiliated Hospital of Naval Medical University, Shanghai, China

**Keywords:** triphenyl phosphate (TPHP), osteoporosis, network toxicology, MAPK signaling pathway, osteoblast

## Abstract

**Objective:**

This study aims to elucidate the molecular mechanisms by which the widely used organophosphate flame retardant and plasticizer, triphenyl phosphate (TPhP), disrupts bone metabolism, highlighting the potential impact of environmental chemicals on bone homeostasis.

**Methods:**

A combined approach of network toxicology and molecular docking was employed to investigate the molecular mechanisms underlying the effects of TPhP on bone metabolism. Potential targets associated with both TPhP and bone metabolism were identified through database searches in ChEMBL, STITCH, GeneCards, and OMIM. A protein–protein interaction (PPI) network was constructed using the STRING database and analyzed with Cytoscape software. Functional enrichment analyses, including Gene Ontology (GO) annotation and Kyoto Encyclopedia of Genes and Genomes (KEGG) pathway analysis, were performed to determine the major pathways involved. Molecular docking was conducted to evaluate the binding affinity between TPhP and key target proteins. Additionally, *in vitro* experiments using MC3T3-E1 osteoblasts were conducted to validate the bioinformatics findings.

**Results:**

78 potential targets related to both TPhP and bone metabolism were identified. STRING and Cytoscape revealed six key proteins: IGF1R, NR3C1, MAP3K1, BRAF, WNK4, and CNR2. GO and KEGG analyses indicated that these targets predominantly function through the MAPK signaling pathway. Molecular docking results demonstrated strong binding affinities between TPhP and key targets, particularly BRAF and WNK4. *In vitro*, TPhP inhibited osteoblast proliferation and migration in a dose-dependent manner and downregulated EMT-related proteins and key target genes via MAPK signaling.

**Conclusion:**

TPhP disrupts bone metabolism by modulating key proteins and pathways, underscoring its potential health risks and the need for further epidemiological and clinical research.

## 1 Introduction

Triphenyl phosphate (TPhP) is a widely used organophosphate flame retardant and plasticizer, which has been detected in various environmental media such as indoor air, dust, soil, and wastewater treatment plant effluent (1, 2). Due to its persistence and difficult degradability, TPhP remains in the environment for an extended period, exhibiting significant bioaccumulation effects, which pose potential threats to human health and environmental safety (3, 4). On November 7, 2024, the European Chemicals Agency (ECHA) included TPhP on the list of substances of very high concern (SVHC), further highlighting its potential hazards.

Numerous studies have shown that TPhP can significantly impact biological health through multiple molecular mechanisms. Research has demonstrated that TPhP has clear developmental toxicity, interfering with cardiac development, skeletal formation, and embryonic development. In the nervous system, TPhP can inhibit acetylcholinesterase activity, disrupt blood-brain barrier integrity, and affect neural function by inducing the release of inflammatory factors and oxidative stress responses (5, 6). In the reproductive system, TPhP can disrupt hormonal balance, impair reproductive cell development, reduce fertility, and pose a carcinogenic risk (7–9). Moreover, TPhP accumulates strongly in the liver and can lead to metabolic disorders by affecting glucose and lipid metabolism and inducing oxidative stress (10–13). Notably, TPhP also exhibits genotoxicity and immunotoxicity, influencing DNA methylation, inducing DNA damage, and disrupting immune responses by modulating immune cell function and inflammatory factor expression (14, 15).

Osteoporosis is a systemic bone disease characterized by low bone mineral density (BMD), bone fragility, and damage to the bone microstructure (16). In recent years, the role of environmental factors in the pathogenesis of osteoporosis has garnered increasing attention. Studies have shown that heavy metal pollutants (such as lead, cadmium, and mercury) can interfere with bone cell function, inhibit osteoblast activity, and promote osteoclast differentiation, leading to increased bone resorption and decreased bone formation, ultimately resulting in osteoporosis (17). Additionally, populations exposed to long-term air pollutants such as PM2.5, PM10, and NO2 exhibit higher risks of osteoporosis and fractures (18). Furthermore, perfluorooctane sulfonate (PFOS) has been shown to be significantly associated with reduced spinal bone mineral density in women, possibly by affecting thyroid hormone and vitamin D metabolism, thereby disrupting bone mineralization (19). However, despite the confirmed multiple mechanisms through which TPhP affects biological health, its impact on bone metabolism has not been systematically studied.

With the rapid development of bioinformatics technology, system-wide analysis based on multi-omics data has provided new research strategies for investigating the mechanisms of environmental pollutants (20). This study aims to integrate resources from databases such as ChEMBL and STITCH, combined with modern bioinformatics methods, to systematically predict and analyze the potential targets of TPhP and its regulatory networks. Through protein-protein interaction network construction, Gene Ontology (GO) functional annotation, and KEGG pathway analysis, this study will provide a deeper understanding of the biological processes and signaling pathways in which these target molecules are involved. Additionally, molecular docking will be used to predict the binding mode of TPhP with key target proteins, and *in vitro* experiments will be conducted to verify the impact of TPhP on osteocyte function. This study will provide new scientific evidence for elucidating the molecular mechanisms of environmental pollutants inducing osteoporosis and has significant theoretical and practical implications for assessing the health risks of TPhP and developing intervention strategies.

## 2 Materials and methods

### 2.1 Cell culture and pharmacological intervention

The murine pre-osteoblastic MC3T3-E1 cell line (passages 5–15) was obtained from Wuhan PriCells Biotechnology Co., Ltd. The cells were routinely cultured in α-MEM complete medium (PriCells, Wuhan) containing 10% premium-grade fetal bovine serum (FBS, PriCells, Wuhan) and 1% penicillin-streptomycin solution (100 U/mL, PriCells, Wuhan). Cultures were maintained at 37°C in a humidified atmosphere with 5% CO_2_. TPhP Treatment: Triphenyl phosphate (TPhP) (Sigma-Aldrich) was dissolved in DMSO (Sigma-Aldrich) to create stock solutions and diluted in α-MEM complete medium to the required concentrations. MC3T3-E1 cells were treated with TPhP at 0 (vehicle control), 10, 20, and 40 μM for subsequent functional assays. For pathway validation experiments, cells were treated with TPhP (40 μM) alone or in combination with the MEK/ERK activator C16-PAF (MCE, at 1 μM) or the MEK inhibitor MEK-IN-6 (MCE 10 μM). Cells were co-incubated for 48 hours before collection for Transwell, CCK-8, or Western blot analysis.

### 2.2 Systematic prediction and analysis of the TPhP action network

The standardized chemical structure and SMILES formula of TPhP were retrieved from the PubChem database (https://pubchem.ncbi.nlm.nih.gov/). A ligand-based multi-target prediction strategy was employed, utilizing the SWISS Target Prediction platform (http://www.swisstargetprediction.ch/) and the SEA-DOCK reverse docking system for high-throughput screening of potential targets. The species parameter was set to “Homo sapiens,” with a prediction probability threshold >0.6. The identified targets were imported into the UniProt database (https://www.uniprot.org/) for standard annotation and functional classification, establishing a dataset of potential TPhP targets.

### 2.3 Multidimensional screening of bone metabolism-related targets

The DisGeNET disease-gene association database (https://www.disgenet.org/) and the TTD therapeutic target database (http://db.idrblab.net/ttd/) were integrated for systematic searches using keywords “osteoporosis,” “bone metabolism,” and “bone remodeling.” Highly relevant genes involved in bone metabolism regulation were screened based on evidence-level scoring (relevance score ≥0.4) to establish a bone metabolism-specific target library. Overlapping genes between TPhP targets and bone metabolism-specific targets were identified using Venn diagram analysis on the BioVenn online platform, forming the candidate key targets for TPhP-mediated regulation of bone metabolism.

### 2.4 Construction and analysis of protein-protein interaction networks

The overlapping genes identified were imported into the STRING database (https://string-db.org/), with a combined confidence threshold >0.4 to construct the protein-protein interaction (PPI) network. Cytoscape software (v3.10.1) was used for network visualization and topological analysis. Functional modules were identified using the MCODE clustering algorithm (degree cutoff = 2, node score cutoff = 0.2, k-core = 2). Core hub genes were determined using 12 ranking algorithms provided by the CytoHubba plugin, including Degree, Betweenness, and Closeness centrality measures.

### 2.5 Multilevel functional enrichment and pathway analysis

Functional enrichment analysis of TPhP’s potential targets was performed using the Metascape platform (https://metascape.org/) and the DAVID annotation system (https://david.ncifcrf.gov/). GO analysis covered three aspects: Biological Processes (BP), Cellular Components (CC), and Molecular Functions (MF). Fisher’s exact test was used to evaluate significance (P < 0.05) with false discovery rate (FDR) correction. KEGG pathway enrichment focused on signaling pathways relevant to bone metabolism regulation, such as the Wnt, BMP, and MAPK pathways.

### 2.6 Structural validation and molecular docking analysis

Molecular docking was performed to investigate the binding characteristics between TPHP and six core target proteins (IGF1R, NR3C1, MAP3K1, BRAF, WNK4, and CNR2) using AutoDock software. The three-dimensional structures of these proteins were obtained from the Protein Data Bank (PDB). Prior to docking, all protein structures were preprocessed by removing water molecules and adding hydrogen atoms. The TPHP structure was energy-minimized before docking simulation. The binding sites were defined based on the surface cavities of each protein. The docking parameters were set to achieve optimal conformational sampling, and the most stable binding conformations were selected based on the lowest binding energy. The binding energy threshold was set at -5 kcal/mol to indicate excellent binding affinity. PyMOL software was utilized to visualize and analyze the three-dimensional configurations of the most stable binding conformations between TPHP and each target protein. The molecular interactions and binding modes were thoroughly examined to evaluate the stability of the protein-ligand complexes.

### 2.7 Core gene identification and validation

Core osteoporosis-related genes were identified using the LASSO (Least Absolute Shrinkage and Selection Operator) regression algorithm. The LASSO regression model was constructed using R software (version 4.1.2) with the “glmnet” package. The penalty parameter λ was determined through 10-fold cross-validation to minimize mean squared error. The optimal λ value was selected when the model achieved the best balance between bias and variance. To validate the screening results, receiver operating characteristic (ROC) curve analysis was performed for both the identified candidate genes and the established osteoporosis model. The area under the curve (AUC) was calculated to evaluate the predictive accuracy of the model, with an AUC threshold of 0.7 considered to indicate significant discriminatory power. The validation process was conducted using the “pROC” package in R.

### 2.8 Wound healing assay

A wound healing assay was conducted to assess the effect of TPhP on MC3T3-E1 cell migration. Cells were cultured to 90–95% confluence, and a uniform scratch was created on the monolayer using a sterile 200 μL pipette tip. Detached cells were gently removed by washing twice with PBS. The cells were then treated with TPhP at 0 (vehicle control), 10, 25, 50, and 100 μM. Images of wound closure at 0 h and 48 h were captured using an inverted microscope equipped with a CCD camera (Nikon Ti2-U, Japan). Image-Pro Plus 6.0 software was used to measure wound areas, and cell migration rates.

### 2.9 Cell proliferation and viability assay

The CCK-8 assay was used to assess the effects of TPhP on cell proliferation. MC3T3-E1 cells in the logarithmic growth phase were seeded into 96-well plates at a density of 1×10^3^ cells/well. After adherence, the cells were treated with TPhP at concentrations ranging from 0 to 100 μM. At 24, 48, 72, 96, and 120 hours, 10 μL of CCK-8 reagent (Beyotime, Wuhan, China) was added to each well and incubated at 37°C for 2 hours in the dark. Absorbance was measured at 450 nm using a microplate reader. Each concentration was tested in triplicate, and the experiments were independently repeated three times.

### 2.10 Transwell invasion and migration assays

A Transwell chamber assay was used to evaluate TPhP’s effects on cell invasion and migration. For the invasion assay, Matrigel (BD, 1:8 dilution) was precoated onto polycarbonate membranes (8 μm pores, Corning) and incubated overnight at 4°C. Cells pre-treated with serum starvation for 12 h were seeded into the upper chamber at 2×10^4^ cells/well in 200 μL serum-free medium containing different concentrations of TPhP. The lower chamber was filled with 600 μL complete medium containing 10% FBS as a chemoattractant. After 24 h, cells on the membrane’s upper surface were gently removed, while invaded cells on the lower surface were fixed with methanol, stained with crystal violet, and counted in five randomly selected fields under a microscope. Migration assays followed the same procedure without Matrigel coating.

### 2.11 Western blot analysis

Cells treated with TPhP were lysed in RIPA buffer containing protease and phosphatase inhibitors (Beyotime, Wuhan) on ice for 30 min. Lysates were centrifuged at 12,000g for 15 min to collect the supernatant. Protein concentration was quantified using a BCA assay, and 30 μg of denatured protein per sample was resolved via SDS-PAGE and transferred onto PVDF membranes (0.45 μm, Millipore) using a semi-dry transfer system. After blocking with 5% BSA in TBST for 2 h, membranes were incubated overnight at 4°C with specific primary antibodies. After washing, HRP-conjugated secondary antibodies (1:5000) were applied for 1 h at room temperature. Signals were developed using enhanced chemiluminescence substrate (Thermo Scientific™ SuperSignal™) and visualized on a ChemiDoc™ MP Imaging System (Bio-Rad). Densitometric analysis was performed using ImageJ software, with β-actin as the loading control. All primary antibodies were obtained from Cell Signaling Technology (CST): p-ERK1/2 (CST, 1:1000); ERK1/2 (CST,1:1000); p-MEK (CST,1:1000); MEK (CST,1:1000); p-P38 (CST,1:1000);P38 (CST,1:1000); p-JNK (CST,1:1000); JNK (CST,1:1000; E-Cadherin (CST,1:1000); N-Cadherin (CST,1:1000); GAPDH (CST,1:1000)

### 2.12 Quantitative PCR

Total RNA was extracted using the TRIzol method (Yeasen, Shanghai), and 1 μg of RNA was reverse-transcribed into cDNA using the PrimeScript™ RT Master Mix (Yeasen, Shanghai). Gene expression was quantified using SYBR Green dye on an ABI QuantStudio™ 3 system. GAPDH was used as an internal control, and relative expression was calculated using the 2^−ΔΔCt^ method. Each sample was tested in triplicate, with experiments independently repeated three times. The primers for all targets are listed in **Table 1**.

**Table 1:** The primers used in the study.

NR3C1-F	CCCAGCATGAGACCAGATGTAAGC
NR3C1-R	CAGAGCACACCAGGCAGAGTTTG
IGF1R-F	TCGACATCCGCAACGACTATC
IGF1R-R	CCAGGGCGTAGTTGTAGAAGAG
MAP3K1-F	GCACGAATGGTTGGAAAGGA
MAP3K1-R	GAGTTGCCAGGAGAAGGACT
BRAF-F	ATCACGGAACAACCC
BRAF-R	GACAACGGAAACCCT
WNK4-F	GTGAAGGCTGCGGAAGACTC
WNK4-R	CTGGGTCTCCATGTCCTCCTT
CNR2-F	ATCACATCCGACTGATCGGC
CNR2-R	GTGAAGGTCATAGTCACGCTG
GAPDH-F	CCTTCATTGACCTCAACTACATGG
GAPDH-R	CTCGCTCCTGGAAGATGGTG

### 2.13 Data processing and statistical analysis

Quantitative data were analyzed and plotted using GraphPad Prism 10.0 software. Results were expressed as mean ± standard deviation (Mean ± SD). Statistical comparisons between two groups were performed using independent sample tt-tests, while one-way analysis of variance (ANOVA) followed by Tukey’s *post hoc* test was used for multiple group comparisons. A two-tailed P-value < 0.05 was considered statistically significant. Significance levels were denoted as follows: *P < 0.05, **P < 0.01, and ***P < 0.001. All experiments were conducted at least three times independently.

## 3 Results

### 3.1 Target identification of TPHP-induced osteoporosis

**Figure 1:**
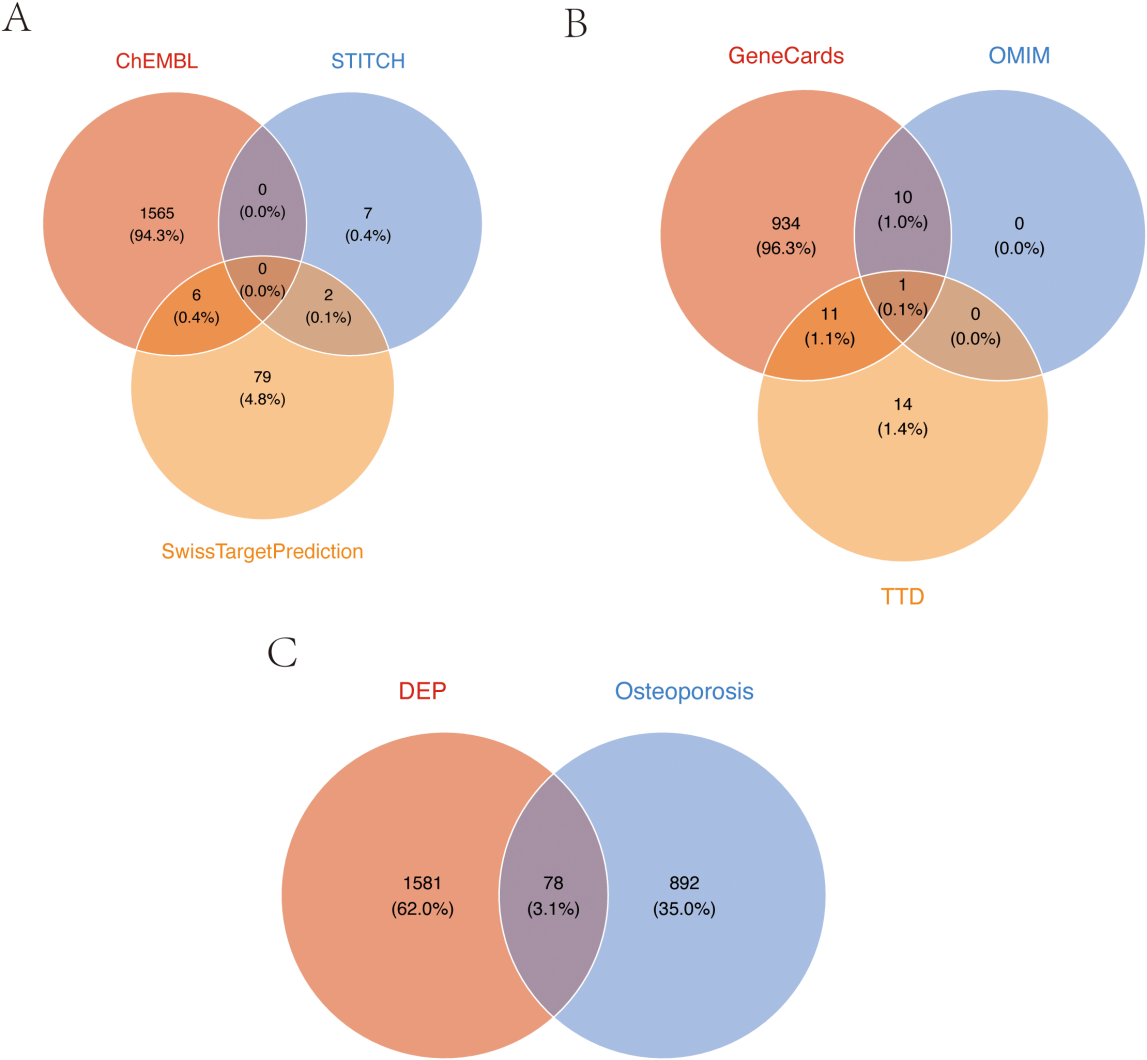
Target screening of TPHP-induced osteoporosis. **(A)** Union set of TPHP-related genes from three databases: CHEMBL, STTCH, and Swiss Target Prediction. **(B)** Union set of osteoporosis-related genes from GeneCard, OMIM, and DisGeNET databases. **(C)** Intersection set between TPHP-related genes and osteoporosis-related genes.

Through comprehensive research, we conducted systematic screening, initially predicting and evaluating TPHP toxicity in PubChem, which revealed active responses to estrogen receptor α (ER) (**Supplementary Figure 1**). It is well established that endogenous estrogen disruption is closely linked to osteoporosis, and previous studies have verified TPHP’s impact on human hormone levels (21, 22). These findings indicate a strong correlation between TPHP and osteoporosis. We initially identified 1,659 potential TPHP-related targets through CHEMBL, STTCH, and Swiss Target Prediction databases (**Figure 1A**). Subsequently, we screened 970 osteoporosis-related genes from GeneCard, OMIM, and DisGeNET databases (**Figure 1B**). After thorough analysis and deduplication, we identified 78 overlapping target molecules, which were considered crucial candidates for TPHP-regulated bone metabolism. The distribution of TPHP and osteoporosis target molecules was visualized using a Venn diagram (**Figure 1C**).

### 3.2 Construction of target molecule interaction network and key gene identification

**Figure 2:**
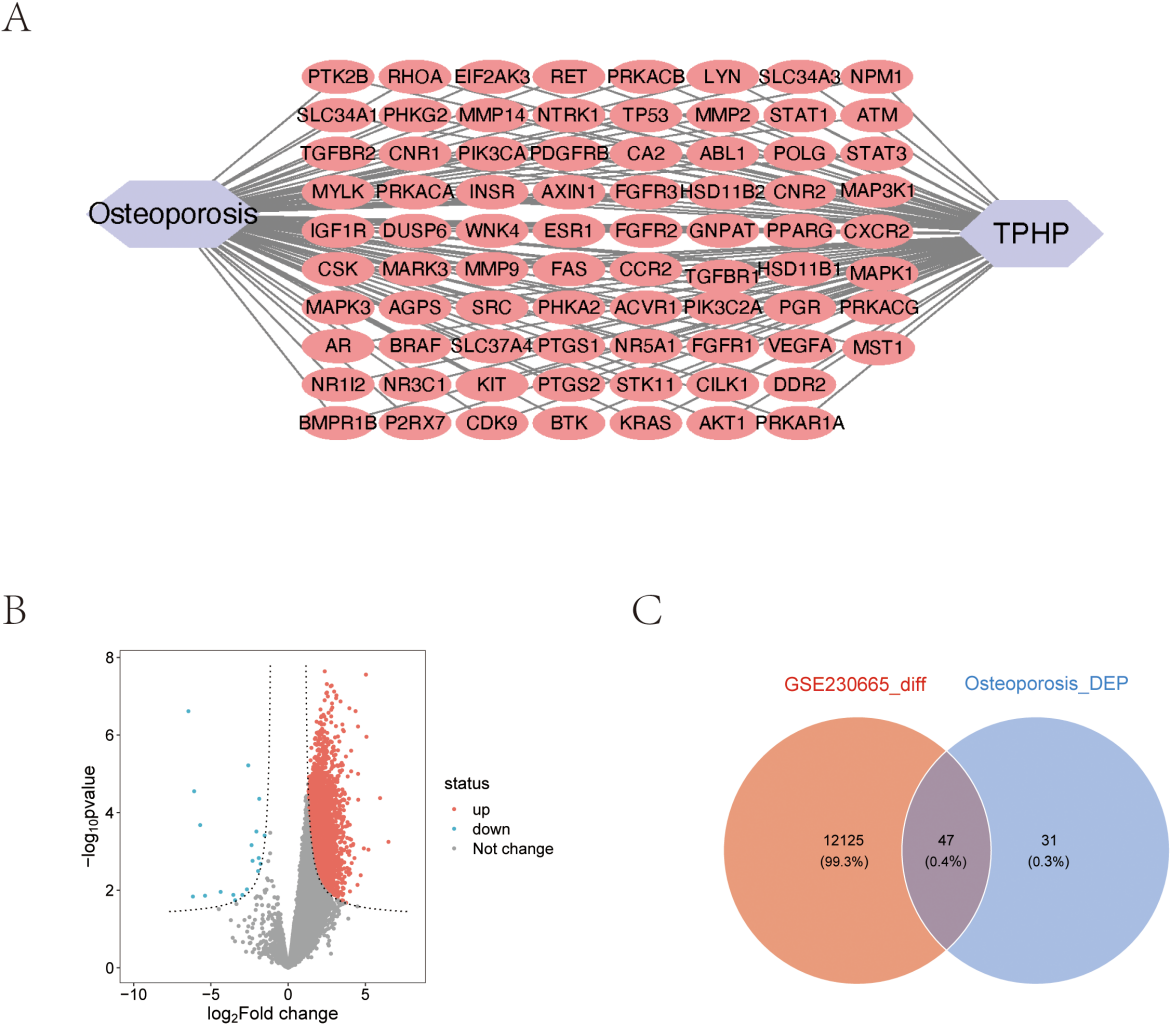
Molecular protein interaction network analysis. **(A)** PPI network analysis of TPHP-influenced osteoporosis-related genes. **(B)** Volcano plot of differentially expressed genes from the GSE230665 dataset. **(C)** Intersection between TPHP-influenced osteoporosis-related genes and differentially expressed genes from the GEO dataset.

Using the STRING database, we constructed a PPI network of osteoporosis-related proteins and visualized their topological features using Cytoscape software. The network diagram clearly illustrated the correlation between relevant proteins, TPHP, and osteoporosis (**Figure 2A**). To further identify key proteins in TPHP-induced osteoporosis, we first conducted an in-depth analysis of osteoporosis-related gene sets in the GEO database to identify differentially expressed genes (**Figure 2B**). By intersecting these differential genes with the aforementioned osteoporosis-related genes, we ultimately identified 47 target molecules significantly influenced by TPHP in osteoporosis (**Figure 2C**).

### 3.3 Pathway enrichment analysis of potential targets

**Figure 3:**
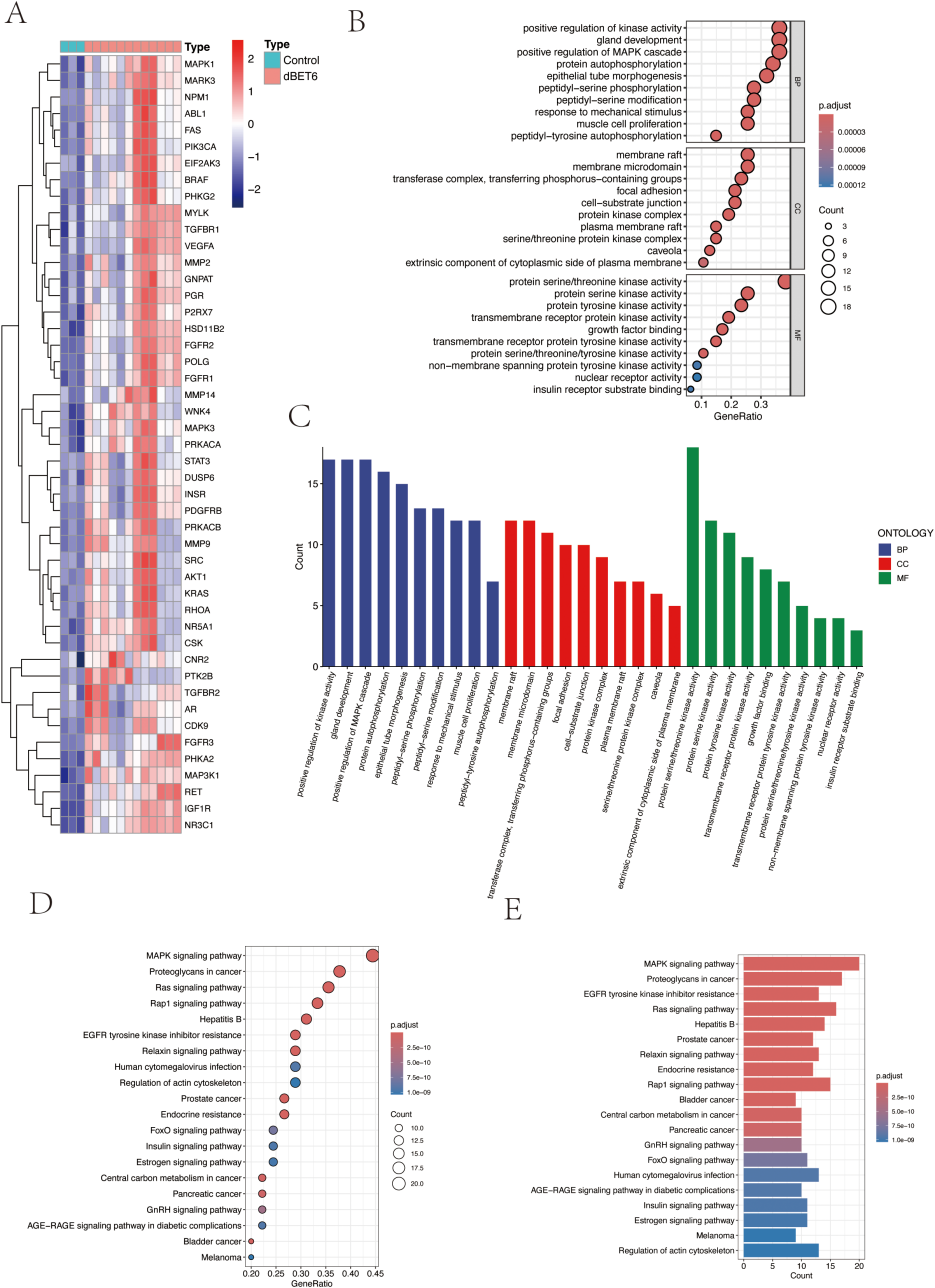
Pathway enrichment analysis of target genes. **(A)** Heatmap of 47 intersection genes between TPHP-influenced osteoporosis-related genes and differentially expressed genes from the GEO dataset. **(B, C)** GO enrichment analysis of target genes, including Biological Process (BP), Cellular Component (CC), and Molecular Function (MF) categories. **(D, E)** KEGG pathway enrichment analysis of target genes, where bubble size and histogram length represent the number of enriched genes.

To investigate the biological functions and signaling pathways of the 47 differentially expressed target proteins (**Figure 3A**), we performed detailed Gene Ontology (GO) functional analysis using the DAVID database, focusing on human-specific aspects. The analysis revealed three major categories: biological processes, cellular components, and molecular functions. We ranked these functions by significance level and visualized the top 10 most significant entries from each category (**Figures 3B, C**). The results indicated that these molecules primarily participate in biological processes including enzyme activity regulation, organ development, signal transduction, and protein modification; in terms of cellular structure, they are mainly distributed in specialized membrane structures, enzyme complexes, and cell junctions; regarding molecular functions, they primarily involve various kinase activities and growth factor binding. Additionally, KEGG pathway enrichment analysis visualized the signaling pathways involving these molecules, which were primarily enriched in MAPK signaling pathway, cellular signal transduction, protein modification, and metabolic regulation pathways (**Figures 3D, E**).

### 3.4 Core gene screening using machine learning algorithms

**Figure 4:**
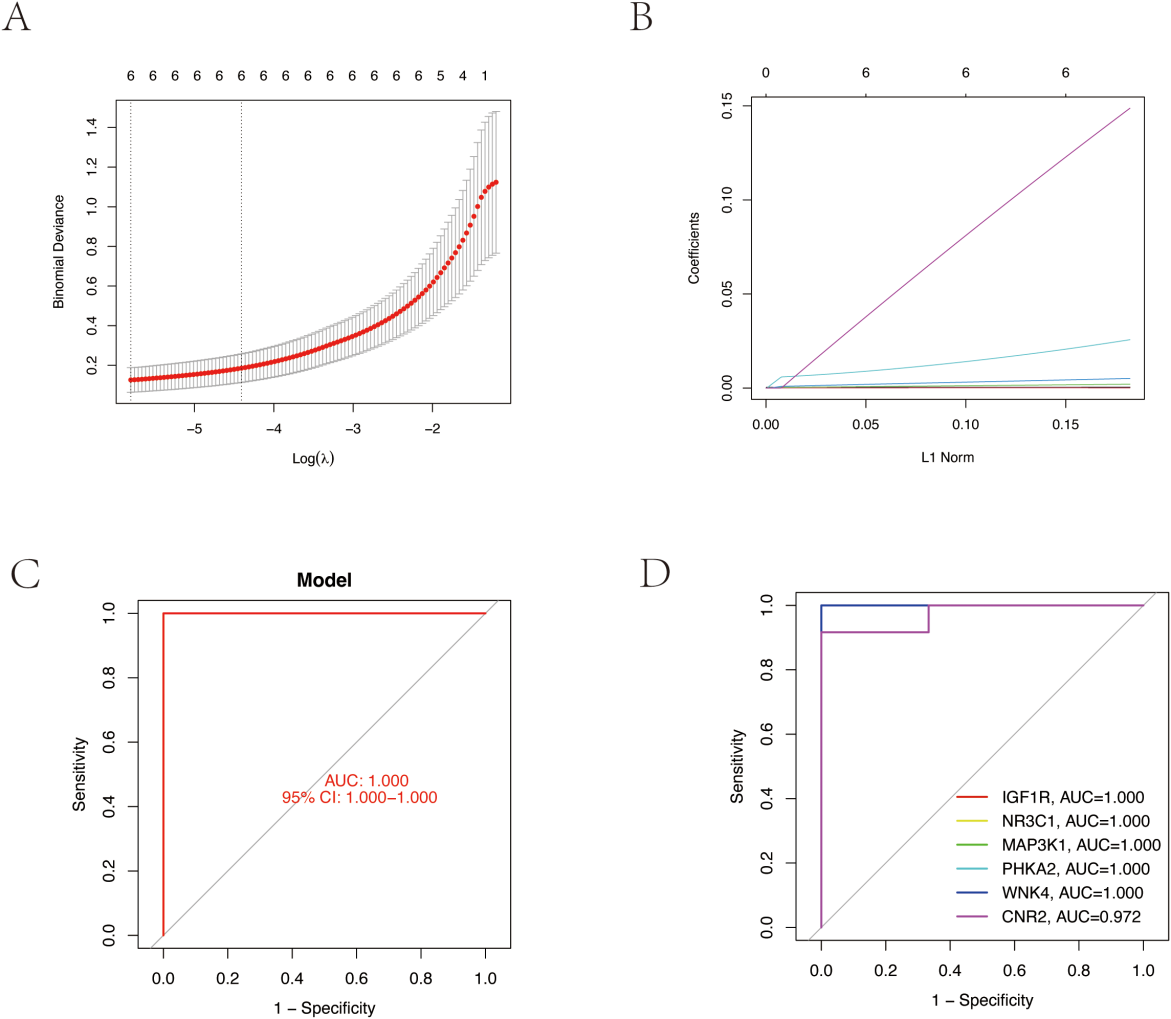
Core target gene screening of TPHP-induced osteoporosis using machine learning algorithms. **(A, B)** Regression curves established by the LASSO regression model, selecting the optimal parameter value λ. Six genes were identified as the most suitable candidate core targets at the lowest point of the curve. **(C)** ROC curve of the model, validating its accuracy. **(D)** ROC curves of six model candidate core genes, validating the scientific validity and accuracy of the selected candidate genes.

We employed LASSO regression machine learning algorithm to screen core osteoporosis-related genes. The LASSO regression algorithm established an osteoporosis-related model, identifying six disease-related candidate core target genes (IGF1R, NR3C1, MAP3K1, BRAF, WNK4, CNR2) (**Figures 4A, B**). To verify the accuracy of the selected candidate genes, we conducted ROC tests on both the candidate genes and the osteoporosis model. The ROC curve results showed AUC values greater than 0.7, confirming the scientific validity of the osteoporosis model construction (**Figures 4C, D**).

### 3.5 Molecular docking of TPHP with core proteins

**Figure 5:**
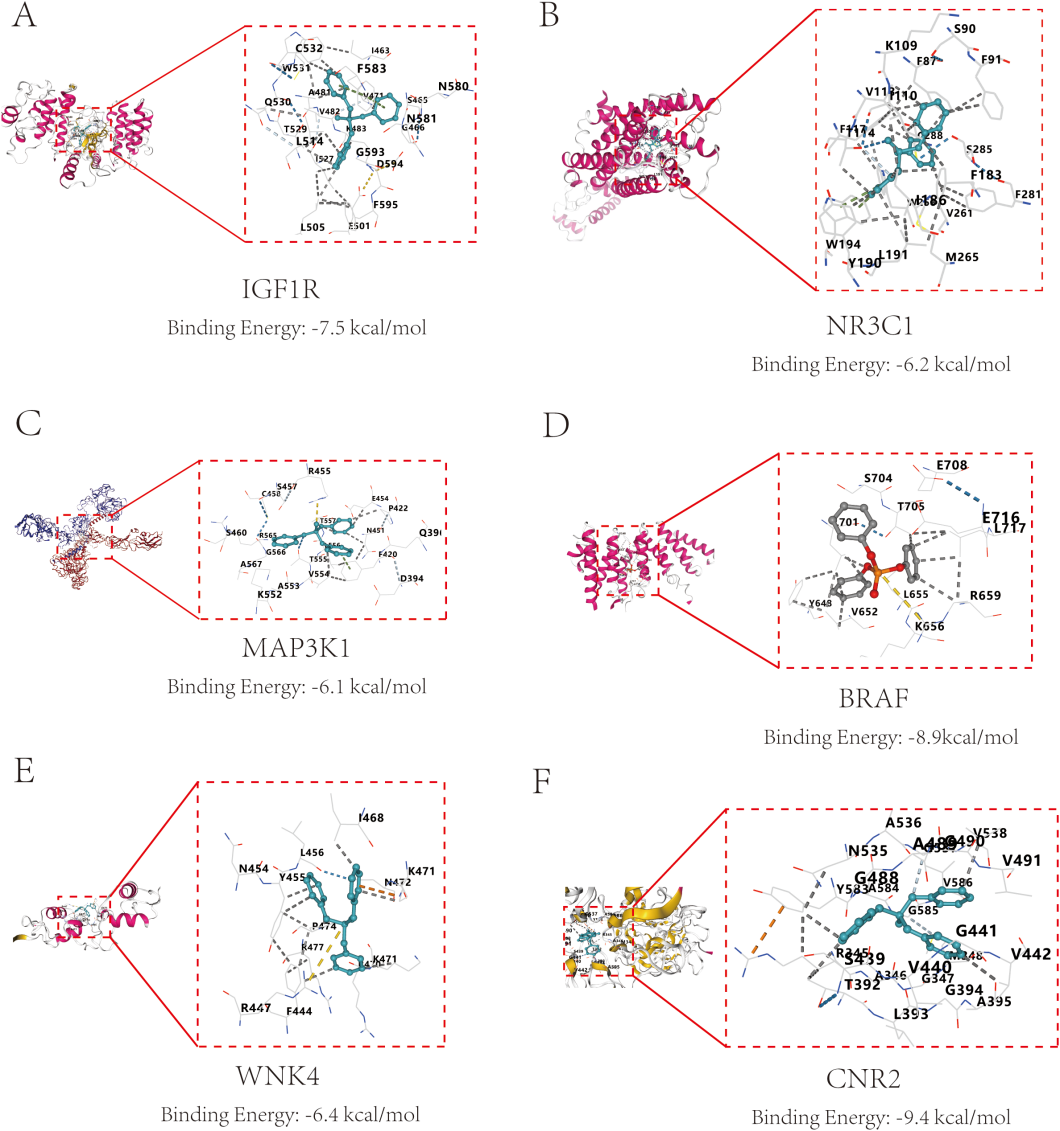
Molecular docking results and binding energies between core proteins and TPHP. **(A)** Binding of TPHP with Insulin-like growth factor 1 receptor (IGF1R), with a binding energy of -7.5 kcal/mol. **(B)** Binding of TPHP with Glucocorticoid receptor (NR3C1), with a binding energy of -6.2 kcal/mol. **(C)** Binding of TPHP with Mitogen-activated protein kinase kinase kinase 1 (MAP3K1), with a binding energy of -6.1 kcal/mol. **(D)** Binding of TPHP with B-Raf proto-oncogene serine/threonine-protein kinase (BRAF), with a binding energy of -8.9 kcal/mol. **(E)** Binding of TPHP with WNK lysine deficient protein kinase 4 (WNK4), with a binding energy of -6.4 kcal/mol. **(F)** Binding of TPHP with Cannabinoid receptor 2 (CNR2), with a binding energy of -9.4 kcal/mol.

To explore the interaction characteristics between TPHP and the six key target proteins (IGF1R, NR3C1, MAP3K1, BRAF, WNK4, and CNR2), we conducted simulation analysis using AutoDock software. It is well known that binding energy below 0 kcal/mol indicates spontaneous binding between receptor and ligand without external energy, while binding energy below -5 kcal/mol indicates excellent binding. The molecular docking results showed that TPHP could tightly bind to the surface cavities of IGF1R, NR3C1, MAP3K1, BRAF, WNK4, and CNR2, with binding energies all below -5 kcal/mol (specifically -7.5, -6.2, -6.1, -8.9, -8.9, -6.4, -9.4 kcal/mol, respectively), indicating high binding stability. Using PyMOL software’s three-dimensional visualization technology, we clearly demonstrated the most stable binding conformations (**Figure 5**), further confirming their tight interactions.

### 3.6 Cellular functional studies

**Figure 6:**
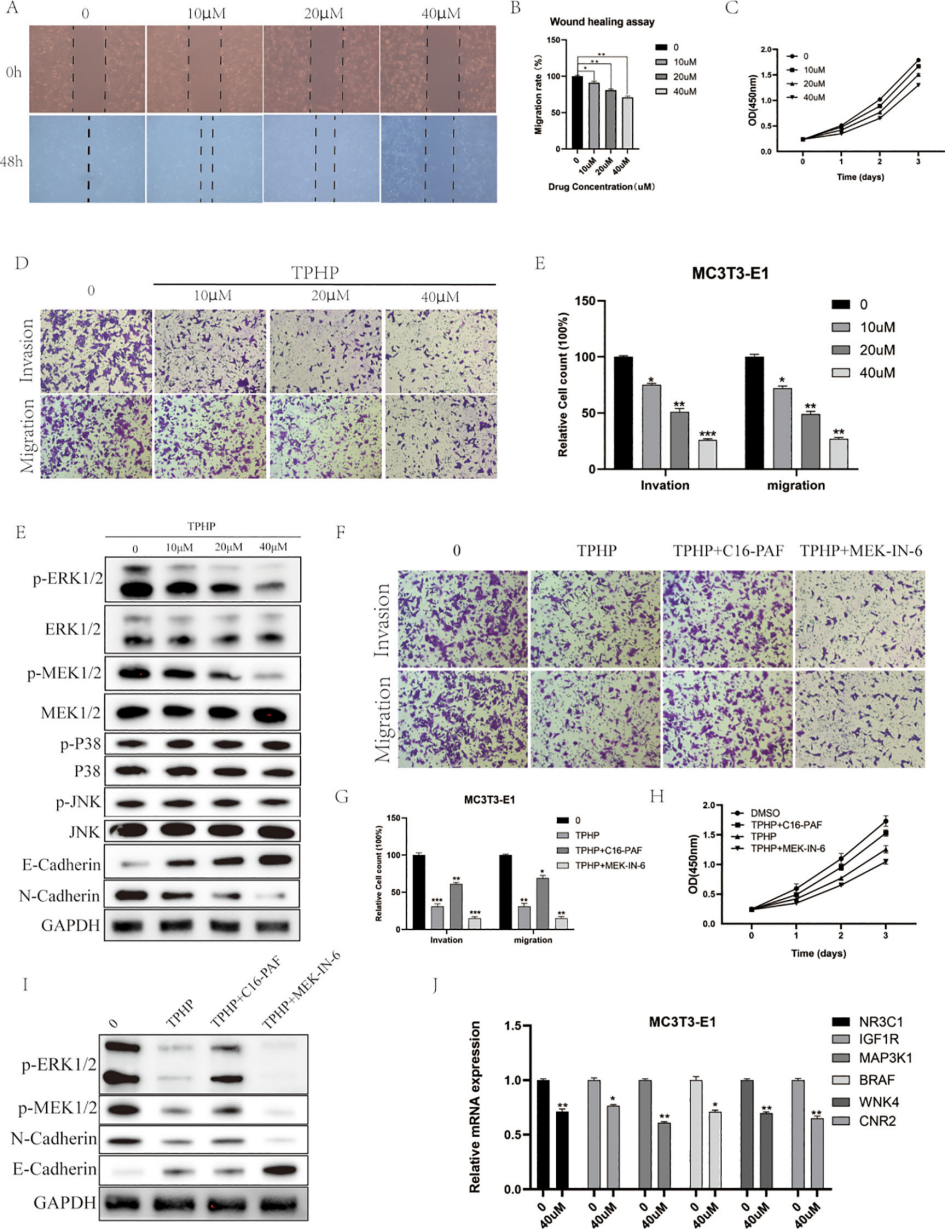
Effects of TPHP on osteoblast function. **(A, B)** TPhP inhibits cell migration (Wound healing assay). **(C)** TPhP reduces cell proliferation (CCK-8 assay). **(D, E)** TPhP suppresses migration and invasion (Transwell assay). **(F)** Western blot showing TPhP effects on MAPK and EMT proteins. **(G, H)** MEK/ERK activator (C16-PAF) rescues TPhP-inhibited migration/invasion **(G)** and proliferation **(H)**, while an inhibitor (MEK-IN-6) does not. **(I)** Western blot confirming MAPK/ERK modulation in rescue experiments. **(J)** qPCR showing TPhP downregulates key target genes.(*P < 0.05, **P < 0.01, ***P < 0.001).

To investigate the direct effects of TPHP on bone cells, we selected MC3T3-E1 mouse osteoblasts as our research model. Cells were exposed to TPHP at concentrations of 0, 10, 20, and 40 μM for 48 hours, and the effects were evaluated through scratch assays and CCK-8 detection. Results showed that TPHP significantly inhibited MC3T3-E1 cell migration in a concentration-dependent manner (**Figures 6A, B**). Cell viability assays further confirmed that TPHP exhibited significant inhibitory effects on cell proliferation, particularly pronounced at higher concentrations (**Figure 6C**). Migration and invasion assays verified TPHP’s inhibitory effect on bone cell migration and invasion capabilities, while Western blot results indicated that TPHP could regulate EMT-related protein expression (**Figure 6D**). Based on previous pathway enrichment analysis, we further investigated whether TPHP affects bone cell function through the MAPK signaling pathway. Next, we explored its molecular mechanisms. Western blot analysis (**Figure 6F**) showed that TPhP treatment led to a significant decrease in the phosphorylation levels of p-MEK and p-ERK1/2 within the MAPK pathway, while p-P38 and p-JNK levels remained unchanged, suggesting that TPhP might specifically inhibit the MEK/ERK signaling axis. Concurrently, TPhP treatment upregulated the expression of the epithelial marker E-Cadherin and downregulated the mesenchymal marker N-Cadherin, indicating a potential impact on the cellular Epithelial-Mesenchymal Transition (EMT) process.

To validate the key role of the MAPK/ERK pathway, we employed a MEK/ERK activator (C16-PAF) and an inhibitor (MEK-IN-6). Results from Transwell (**Figure 6G**) and CCK-8 (**Figure 6H**) assays demonstrated that C16-PAF significantly reversed the TPhP-induced inhibition of cell invasion/migration and proliferation, indicating that reactivating the MEK/ERK pathway could “rescue” the negative effects of TPhP. In contrast, the MEK inhibitor MEK-IN-6 failed to reverse TPhP’s effects. Correspondingly, Western blot results (**Figure 6I**) confirmed that C16-PAF restored the TPhP-suppressed phosphorylation levels of p-ERK1/2 and p-MEK1/2 and reversed the changes in E-Cadherin and N-Cadherin expression. Finally, we used qPCR to examine the effect of TPhP on the expression of the six previously identified key target genes. The results showed that, compared to the control group, 40 μM TPhP treatment significantly downregulated the mRNA levels of NR3C1, IGF1R, MAP3K1, BRAF, WNK4, and CNR2 (**Figure 6J**).

In summary, the results presented in **Figure 6** visually demonstrate that TPhP impairs the proliferation, migration, and invasion functions of MC3T3-E1 osteoblasts by inhibiting the MAPK/ERK signaling pathway and downregulating associated gene expression.

## 4 Discussion

This study systematically explored the potential effects of triphenyl phosphate (TPhP) on bone metabolism using network toxicology and molecular docking techniques. First, we integrated information from databases such as ChEMBL, STITCH, GeneCards, and OMIM, and combined this data with the STRING platform and Cytoscape software to construct a network of potential target interactions. Through this analysis, we identified six key targets, including IGF1R, NR3C1, MAP3K1, BRAF, WNK4, and CNR2, which revealed the core mechanisms by which TPhP affects bone metabolism and provided important clues for future research. Further, at the cellular functional level, we confirmed for the first time that TPhP significantly inhibits the proliferation and migration abilities of osteoblasts (MC3T3-E1 cells) in a dose-dependent manner. Specifically, exposure to high concentrations of TPhP led to significant inhibition of both cell migration and proliferation. Moreover, Western blot analysis indicated that TPhP regulates the expression of proteins related to epithelial-mesenchymal transition (EMT). qPCR results showed a significant downregulation of the expression levels of the six key target genes after TPhP treatment, which was highly consistent with the bioinformatics predictions. Through this multi-layered integrated approach, our study not only elucidated the potential molecular mechanisms of TPhP on bone metabolism but also provided a solid theoretical foundation for further investigation into its toxic effects.

Previous studies have highlighted that endocrine disruption and reproductive toxicity are significant effects of TPhP. Research has shown that TPhP can interfere with hormonal balance by binding to estrogen receptors or blocking related signaling pathways. For instance, in animal models, exposure to TPhP results in reduced sperm motility and abnormal morphology, which in turn affects reproductive ability (23). In females, TPhP affects ovarian development, decreases the expression of vitellogenin, and significantly weakens reproductive capability (24). Additionally, TPhP has been confirmed to activate tumor-related signaling pathways, promoting the proliferation of endometrial cancer cells, suggesting its potential carcinogenic risk (8). This aligns with our findings, as we identified 78 target molecules related to TPhP and osteoporosis, and the constructed PPI network, along with differential gene analysis, further focused on 47 significantly associated molecules. Pathway enrichment analysis indicated that TPhP can indirectly affect hormonal balance closely related to osteoporosis by actively interacting with estrogen receptor α (ERα). Estrogen plays a crucial role in bone remodeling, particularly in regulating the balance between osteoblasts and osteoclasts. Studies have shown that estrogen promotes osteoblast differentiation via the classic ERα-mediated gene transcription pathway, while inhibiting osteoclast formation and activity, thus reducing bone resorption (25). Other studies have shown that estrogen can inhibit the expression of RANKL and enhance the secretion of OPG, which suppresses osteoclast maturation while maintaining bone density (26). The significant decline in estrogen levels in peri-menopausal women is considered a major cause of osteoporosis, accompanied by accelerated bone resorption and degradation of trabecular structure (27, 28). Increasing evidence suggests that estrogen plays an immune-regulatory role in maintaining the bone microenvironment, with studies showing that estrogen regulates macrophage and T-cell function, further affecting bone metabolism (29). Therefore, any exogenous chemicals that disrupt estrogen signaling pathways could significantly impact bone metabolism.

Our molecular docking results revealed stable binding characteristics between TPhP and the six key target proteins, with binding energies all below -5 kcal/mol, particularly showing a binding energy of -8.9 kcal/mol with BRAF and WNK4, indicating that these interactions may have biological significance. These results not only validate the key targets identified through bioinformatics methods but also aid in understanding the molecular mechanisms by which TPhP affects bone metabolism. Among the six targets, BRAF and MAP3K1 are key regulators of the MAPK signaling pathway, which is consistent with previous research as the MAPK pathway plays a critical role in osteoblast proliferation, differentiation, and apoptosis resistance (30, 31). Abnormal activation or inhibition of BRAF could directly interfere with osteogenesis, while WNK4, a kinase primarily regulating ion transport and cellular homeostasis, shows a strong binding affinity with TPhP, suggesting that TPhP may promote osteoporosis by disrupting ion balance (32). Notably, the involvement of CNR2 provides a new direction for exploring the mechanism by which TPhP induces osteoporosis. CNR2, as a core receptor in the endogenous cannabinoid system, has been shown to play an active role in osteoblast differentiation and the regulation of bone resorption (33, 34). Our molecular docking results show that TPhP has a high binding affinity with CNR2, which may suppress bone formation and accelerate bone resorption by interfering with CNR2 function. This finding is consistent with recent studies on the role of the endogenous cannabinoid system in bone metabolism, providing new targets for exploring the mechanisms by which environmental pollutants disrupt bone metabolism. *In vitro* experiments further validated the direct inhibitory effect of TPhP on osteoblasts. Scratch assays and CCK-8 assays showed that TPhP significantly inhibited the migration and proliferation abilities of MC3T3-E1 osteoblasts in a concentration-dependent manner. This inhibitory effect may be mediated through the MAPK signaling pathway, specifically as our Western blot results showed a significant downregulation of ERK phosphorylation in the MAPK pathway after TPhP treatment, while JNK and p38 pathways were not significantly altered; concurrently, qPCR analyses also showed downregulation of key genes related to upstream MAPK signaling. These findings collectively point toward TPhP interfering with osteoblast function by specifically affecting the MAPK/ERK signaling axis. The changes in the expression of EMT-related proteins particularly suggest that TPhP may further reduce osteoblast functional activity by affecting cell adhesion.

To further clarify the molecular mechanisms of TPhP on bone metabolism, experimental validation of the computational network analysis results is essential. However, there are some limitations in the current study. First, existing toxicity assessments primarily focus on cellular levels and have not fully considered the complex molecular disturbances that may occur *in vivo*, nor their conservation across different biological systems. Second, the causal relationship between TPhP exposure and bone metabolic abnormalities has not been clearly verified in animal models. The acute, high-dose exposure model used in the experiments may not accurately reflect the long-term, low-dose exposure scenario in humans due to environmental pollution and consumer product contact with TPhP. Moreover, the complexity of bone metabolic diseases, especially in aging populations, is often influenced by long-term, multifactorial factors, suggesting that short-term animal experiments may not fully reveal the cumulative effects of chronic low-dose TPhP exposure on human bone health.

In conclusion, this study provides strong evidence for the multi-layered mechanisms by which TPhP interferes with bone metabolism, including hormonal disruption, signaling pathway dysregulation, and ion homeostasis disturbance. These findings not only deepen our understanding of the toxic effects of TPhP on bone health but also highlight potential molecular targets for mitigating its adverse impacts. Future studies should focus on *in vivo* validation of these mechanisms and explore intervention strategies to address TPhP exposure.

## Data Availability

The original contributions presented in the study are included in the article/**Supplementary Material**. Further inquiries can be directed to the corresponding authors.
